# Impact of schizophrenia on coronary artery bypass grafting outcomes: a nationwide analysis

**DOI:** 10.1097/MS9.0000000000005349

**Published:** 2026-07-30

**Authors:** Chinenye Cynthia Odo, Adeleke Oluwaseun Dorcas, Andrew Greek, Enyioma Nwogwugwu, Chukwuebuka Asogwa, Emmanuel Daniel, Oluwamisimi Abib, Oluwaremilekun Zeth Tolu-Akinnawo, Chinelo Eneh, Toluwalase Awoyemi

**Affiliations:** a Department of Internal Medicine, University of Pittsburgh, Pittsburgh PA, USA; b Faculty of Medicine, Sumy State University, Sumy, Ukraine; c Department of Internal Medicine, Kansas City University, Joplin, MO, USA; d Department of Internal Medicine, Lincoln Medical and Mental Health Center, Bronx NY, USA; e Department of Medicine and Surgery, College of Medicine, University of Ibadan, Ibadan, Nigeria; f Department of Internal Medicine, Trinity Health, Ann Arbor, MI, USA; g Department of Internal Medicine, Piedmont Athens Regional, Athens, Georgia, USA; h Department of Internal Medicine, Meharry Medical College, Nashville, TN, USA; i Department of English, Florida State University, Tallahassee, Florida, USA; j Department of Cardiology, Mass General Brigham, Harvard Medical School, Boston, Massachusetts, USA

**Keywords:** cardiac surgery outcomes, coronary artery bypass grafting, health disparities, perioperative mortality, schizophrenia, serious mental illness

## Abstract

**Background:**

Schizophrenia is associated with an elevated cardiovascular disease burden and mortality. However, outcomes for patients with schizophrenia undergoing coronary artery bypass grafting (CABG) remain poorly characterized. We investigated perioperative outcomes, resource utilization, and discharge disposition in this vulnerable population.

**Methods:**

Using the National Inpatient Sample (2017–2020), we identified 773 925 adult CABG admissions, including 2250 patients with schizophrenia and 771 675 without. The primary outcome was in-hospital mortality. Secondary outcomes included length of stay, hospital charges, post-operative complications, and discharge disposition. Multivariable logistic and linear regression models were adjusted for demographics, comorbidities, and hospital characteristics.

**Results:**

Patients with schizophrenia were younger (59.6 vs 66.3 years, *P* < 0.001), had a higher comorbidity burden (Charlson index 3.1 vs 2.7, *P* < 0.001), and a greater prevalence of diabetes (58.0% vs 51.1%, *P* < 0.0001), tobacco use (41.6% vs 18.4%, *P* < 0.001), and prior myocardial infarction (21.6% vs 17.5%, *P* < 0.001). After adjustment, schizophrenia was associated with increased odds of in-hospital mortality of borderline statistical significance (adjusted OR 1.30, 95% CI 1.001–1.69, *P* = 0.049), prolonged hospitalization (1.74 additional days, 95% CI 1.40–2.04, *P* < 0.0001), and discharge to skilled nursing facilities (adjusted OR 1.74, 95% CI 1.33–2.27, *P* < 0.0001). Post-operative infection, stroke, and bleeding rates were similar between groups.

**Conclusions:**

Schizophrenia was associated with increased odds of in-hospital mortality of borderline statistical significance (adjusted OR 1.30, 95% CI 1.001–1.69, *P* = 0.049), prolonged hospitalization, and a greater need for post-acute skilled nursing care following CABG. These findings highlight the need for integrated perioperative psychiatric care, optimized perioperative protocols, and structured discharge planning to improve outcomes and ensure equitable care for patients with serious mental illness undergoing cardiac surgery.

## Introduction

Schizophrenia affects approximately 1% of the global population and is associated with a substantially reduced life expectancy, with patients dying 15–20 years earlier than the general population^[^1–3^]^. Cardiovascular disease represents the leading cause of premature mortality in this population, accounting for up to 40% of deaths^[^4,5^]^ Multiple factors contribute to this elevated cardiovascular risk, including higher rates of traditional risk factors (diabetes, hypertension, dyslipidemia, tobacco use), antipsychotic medication effects, sedentary lifestyle, poor diet, and systemic barriers to accessing preventive cardiovascular care^[^6–8^]^.

HIGHLIGHTS

- Schizophrenia is independently associated with increased in-hospital mortality after coronary artery bypass grafting (CABG).
- Patients with schizophrenia experience significantly longer hospital stays following CABG.
- Schizophrenia is linked to a higher likelihood of being discharged to skilled nursing facilities.
- Post-operative complication rates are largely similar after risk adjustment.
- Substantial demographic and socioeconomic disparities exist among CABG patients with schizophrenia.

Despite this well-established cardiovascular disease burden, outcomes for patients with schizophrenia undergoing major cardiovascular interventions remain poorly characterized. Prior research has documented disparities in access to advanced cardiac procedures, with patients with serious mental illness being less likely to receive revascularization despite similar disease severity^[^9,10^]^. When cardiac interventions are performed, limited data suggest worse outcomes, though small sample sizes or single-center experiences have constrained most studies^[^11–13^]^.

Coronary artery bypass grafting (CABG) remains a cornerstone of revascularization for patients with complex multivessel coronary artery disease and left main disease. Understanding outcomes in patients with schizophrenia is critical for several reasons: informing perioperative risk stratification, guiding resource allocation, identifying opportunities for quality improvement, and ensuring equitable care delivery. Prior studies examining psychiatric comorbidities and cardiac surgery outcomes have produced inconsistent findings, with most focusing on depression or anxiety rather than schizophrenia specifically^[^14,15^]^.

Using a large, nationally representative database, we sought to comprehensively evaluate perioperative outcomes, resource utilization, and discharge patterns for patients with schizophrenia undergoing CABG. We hypothesized that schizophrenia would be independently associated with increased mortality, prolonged hospitalization, and greater post-acute care needs after adjustment for demographic and clinical factors. This analysis is compliant with the TITAN Guidelines 2025[16].

## Methods

### Data source and study population

This retrospective cohort study utilized the National Inpatient Sample (NIS) from 2017 to 2020, maintained by the Healthcare Cost and Utilization Project of the Agency for Healthcare Research and Quality (AHRQ). The NIS represents the largest all-payer inpatient database in the United States, approximating a 20% stratified sample of all US community hospital discharges. We applied discharge weights provided by AHRQ to generate national estimates.

Adult patients (≥18 years) undergoing CABG were identified using ICD-10-PCS procedure codes for CABG procedures. Schizophrenia was identified using ICD-10-CM diagnosis codes (F20.x). Patients were categorized into schizophrenia and non-schizophrenia cohorts. Patients under 18 years of age were excluded, as were admissions with missing data for key demographic or outcome variables (affecting fewer than 1% of records) and elective admissions transferred from another acute care facility.

### Outcomes

The primary outcome was in-hospital mortality. Secondary outcomes included: (1) length of stay (days); (2) total hospital charges; (3) post-operative complications (infection, stroke, bleeding, cardiovascular complications, thrombosis); and (4) discharge disposition (routine home discharge, home health care, skilled nursing facility, short-term hospital transfer, or discharge against medical advice).

### Covariates

Demographic variables included age, sex, race/ethnicity (Caucasian, Black/African American, Hispanic, Asian/Pacific Islander, Native American, other), primary insurance (Medicare/Medicaid [public], private, other), and median household income by ZIP code quartile. Hospital characteristics included bed size (small, medium, large), teaching status (rural, urban non-teaching, urban teaching), and geographic region. Clinical covariates included the Charlson Comorbidity Index and specific comorbidities: hypertension, diabetes mellitus, ischemic heart disease, obesity, tobacco use, dyslipidemia, atrial fibrillation, prior myocardial infarction, and prior cardiac arrest.

### Statistical analysis

Descriptive statistics summarized baseline characteristics and outcomes. Continuous variables were reported as mean ± standard deviation and compared using independent samples t-tests (for normally distributed data) or Mann–Whitney U tests (for skewed distributions). Categorical variables were reported as frequencies and percentages and compared using Pearson χ^2^ or Fisher’s exact tests (for expected cell counts <10).

Multivariable regression models assessed independent associations between schizophrenia and outcomes. Logistic regression estimated adjusted odds ratios for binary outcomes, while linear regression estimated adjusted mean differences for continuous outcomes. Models included all covariates significantly associated with outcomes in univariable analysis (*P* < 0.10), as well as clinically relevant confounders regardless of significance. Model discrimination was assessed using C-statistics for logistic models. Two-sided *P*-values < 0.05 indicated statistical significance. Analyses were performed using SAS Studio 3.8.2 and SPSS version 29.0.

## Results

### Study population and baseline characteristics

**Figure 1.**
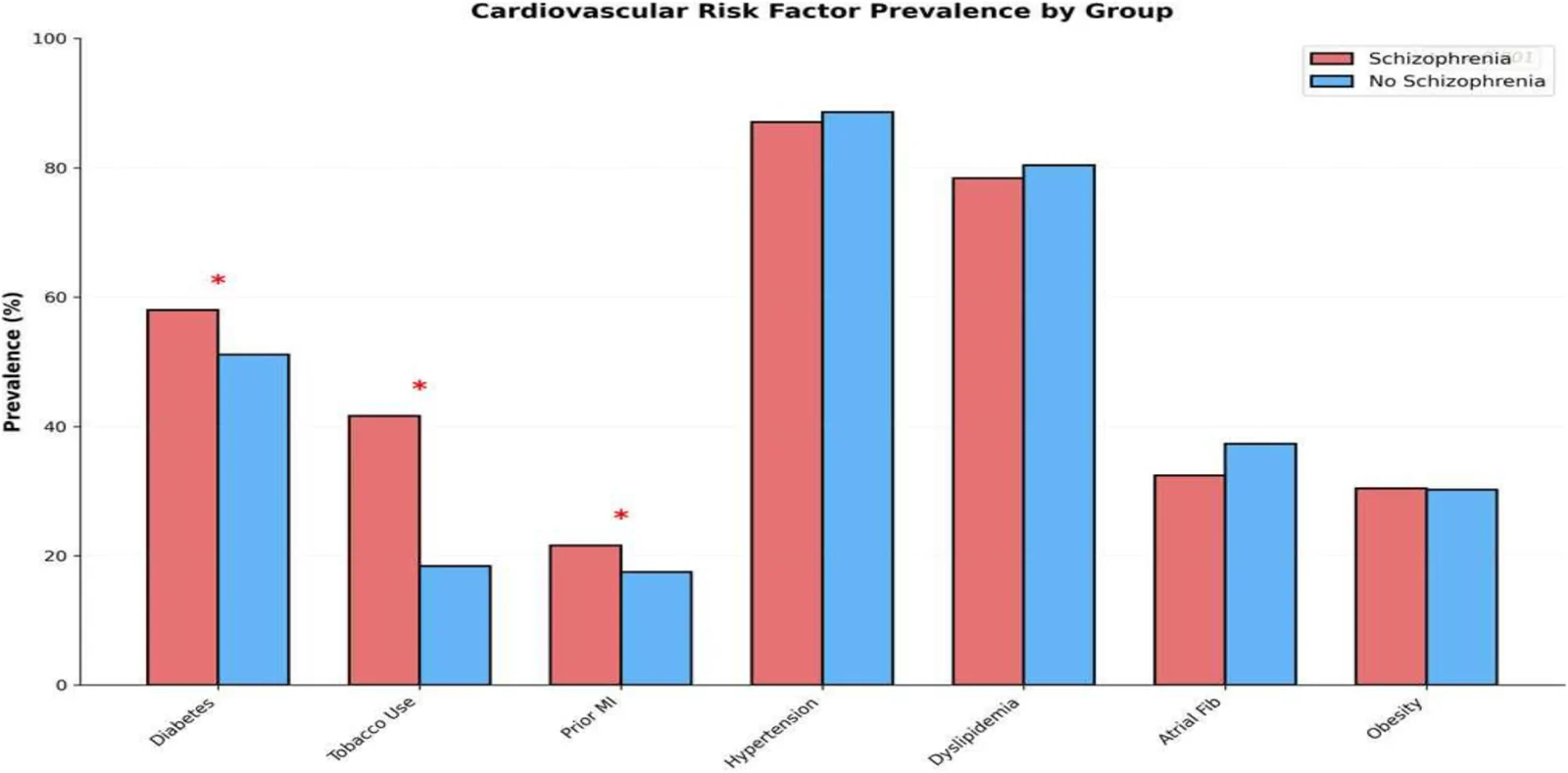
Prevalence of selected cardiovascular risk factors and comorbidities among patients undergoing CABG, stratified by schizophrenia status. Asterisks (*) denote statistically significant between-group differences (*P* < 0.05). CABG, coronary artery bypass grafting.

Among 773 925 weighted CABG admissions from 2017 to 2020, 2250 patients (0.29%) had documented schizophrenia. Patients with schizophrenia were significantly younger (mean age 59.6 vs 66.3 years, *P* < 0.001) and had a higher comorbidity burden (Charlson index 3.1 vs 2.7, *P* < 0.001). The schizophrenia cohort demonstrated a higher prevalence of several cardiovascular risk factors, including diabetes mellitus (58.0% vs 51.1%, *P* < 0.0001), tobacco use (41.6% vs 18.4%, *P* < 0.001), and prior myocardial infarction (21.6% vs 17.5%, *P* < 0.001). Conversely, schizophrenia patients had lower rates of hypertension (87.1% vs 88.6%, *P* = 0.0328), dyslipidemia (78.4% vs 80.4%, *P* = 0.0210), and atrial fibrillation (32.4% vs 37.3%, *P* < 0.001) (Fig. 1).

Significant demographic differences were observed. The schizophrenia group had higher proportions of Black/African American (19.8% vs 7.0%) and Hispanic (10.6% vs 7.7%) patients compared to non-schizophrenia patients, who were predominantly Caucasian (78.6% overall). Schizophrenia patients were more likely to have public insurance (80.9% vs 63.9%, *P* < 0.001) and reside in lower-income ZIP codes, with 42.7% in the lowest income quartile compared to 27.7% of non-schizophrenia patients (*P* < 0.001). Most patients in both groups underwent CABG at large, urban teaching hospitals (Table 1).

**Table 1:** Baseline characteristics and comorbidities of patients undergoing CABG by schizophrenia status.

Variable	Schizophrenia (*n* = 2250)	No Schizophrenia (*n* = 771 675)	*P*-value
**Demographics**			
Age, mean ± SD (years)	59.6 ± 21.6	66.3 ± 22.3	<0.001
Male sex, *n* (%)	1675 (74.4)	582 945 (75.5)	0.226
Race/ethnicity, *n* (%)			<0.001
Caucasian	1405 (64.6)	585 885 (78.6)	
Black/African American	430 (19.8)	52 155 (7.0)	
Hispanic	230 (10.6)	57 280 (7.7)	
Other	110 (5.0)	50 155 (6.7)	
Insurance type, *n* (%)			<0.001
Public (Medicare/Medicaid)	1820 (80.9)	492 405 (63.9)	
Private	195 (8.7)	230 555 (29.9)	
Other	235 (10.4)	47 580 (6.2)	
**Comorbidities**			
Charlson Comorbidity Index, mean ± SD	3.1 ± 4.1	2.7 ± 4.4	<0.001
Hypertension, *n* (%)	1960 (87.1)	683 290 (88.6)	0.033
Diabetes mellitus, *n* (%)	1305 (58.0)	393 960 (51.1)	<0.0001
Ischemic heart disease, *n* (%)	2200 (97.8)	756 905 (98.1)	0.287
Obesity, *n* (%)	685 (30.4)	231 645 (30.2)	0.660
Tobacco use, *n* (%)	935 (41.6)	141 995 (18.4)	<0.001
Dyslipidemia, *n* (%)	1765 (78.4)	620 275 (80.4)	0.021
Atrial fibrillation, *n* (%)	730 (32.4)	287 735 (37.3)	<0.001
Prior myocardial infarction, *n* (%)	485 (21.6)	135 155 (17.5)	<0.001
Prior cardiac arrest, *n* (%)	25 (1.1)	3770 (0.5)	<0.001

Data presented as *n* (%) for categorical variables and mean ± SD for continuous variables. SD, standard deviation.

### In-hospital mortality

Unadjusted in-hospital mortality was numerically higher in the schizophrenia group (2.9% vs 2.5%, *P* = 0.2433), though this difference did not reach statistical significance. After multivariable adjustment for demographics, comorbidities, and hospital characteristics, schizophrenia was associated with increased in-hospital mortality (adjusted OR 1.30, 95% CI 1.001–1.69, *P* = 0.049), though the confidence interval narrowly excluded the null, and this finding should be interpreted with caution. Other independent predictors of mortality included advanced age, higher Charlson index, female sex, racial/ethnic minority status, and atrial fibrillation. Protective factors included diabetes, ischemic heart disease, dyslipidemia, prior MI, and higher household income (Tables 2–3).

**Table 2:** Clinical outcomes following CABG by schizophrenia status.

Outcome	Schizophrenia (*n* = 2250)	No schizophrenia (*n* = 771 675)	Unadjusted *P*-value	Adjusted OR (95% CI)	Adjusted *P*-value
In-hospital mortality, *n* (%)	65 (2.9)	19 315 (2.5)	0.243	1.30 (1.001–1.69)	0.049
Length of stay, mean ± SD (days)	12.3 ± 16.8	9.9 ± 16.9	<0.001	1.74 days^a^ (1.40–2.04)	<0.0001
Hospital charges, mean ± SD ($)	271 496	239 616	0.001	$17 223^a^	0.075
Post-operative infection, *n* (%)	65 (2.9)	16 115 (2.1)	0.008	1.27 (0.98–1.63)	0.069
Post-operative stroke, *n* (%)	15 (0.7)	5275 (0.7)	0.923	1.04 (0.62–1.73)	0.894
Post-operative bleeding, *n* (%)	5 (0.2)	2725 (0.4)	0.296	0.77 (0.32–1.85)	0.551

^a^ Adjusted difference (linear regression). OR, odds ratio; CI, confidence interval; SD, standard deviation.

**Table 3:** Discharge disposition following CABG by schizophrenia status.

Discharge disposition	Schizophrenia *n* (%)	No schizophrenia *n* (%)	Adjusted OR (95% CI)	*P*-value
Routine home	550 (24.4)	287 605 (37.3)	0.38 (0.29–0.51)	<0.0001
Home health care	775 (34.4)	297 695 (38.6)	0.56 (0.43–0.74)	<0.001
Skilled nursing facility/ Intermediate care facility	845 (37.6)	160 255 (20.8)	1.74 (1.33–2.27)	<0.0001
Short-term hospital	15 (0.7)	5565 (0.7)	0.67 (0.38–1.19)	0.172
Against medical advice	0 (0.0)	940 (0.1)	—	—

OR, odds ratio; CI, confidence interval. Adjusted for age, sex, race/ethnicity, insurance, hospital characteristics, and comorbidities.

### Length of stay and hospital charges

**Figure 2.**
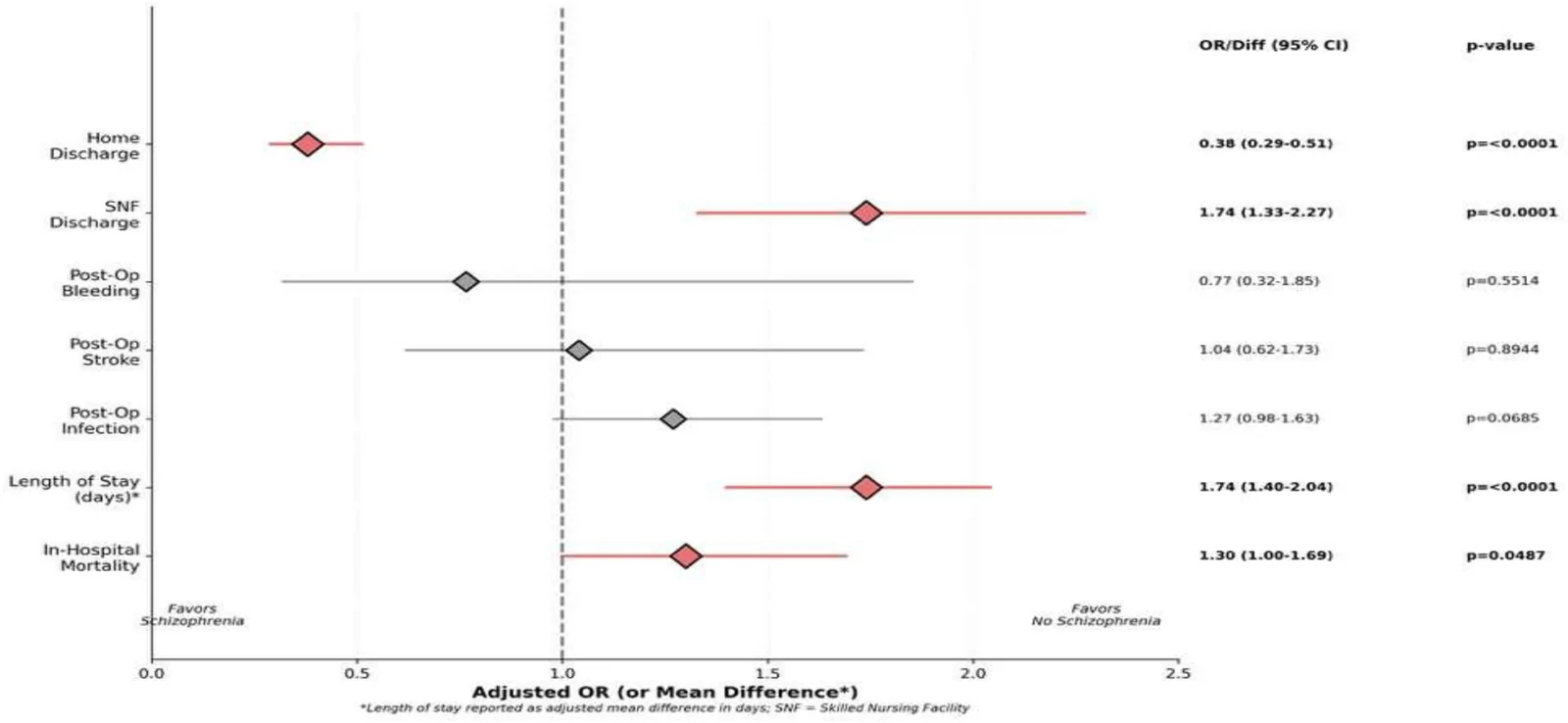
Adjusted odds ratios and mean differences for perioperative outcomes among patients with versus without schizophrenia undergoing CABG. Points represent adjusted estimates; horizontal lines represent 95% confidence intervals. Red diamonds indicate statistically significant associations (*P* < 0.05); gray diamonds indicate non-significant associations. Length of stay is reported as the adjusted mean difference in days. OR, odds ratio; CI, confidence interval; SNF, skilled nursing facility.

Mean length of stay was significantly longer for schizophrenia patients (12.3 vs 9.9 days, *P* < 0.001) (Fig. 2). This difference remained significant after adjustment, with schizophrenia patients staying 1.74 additional days (95% CI: 1.40–2.04, *P* < 0.0001). Mean hospital charges were higher in the schizophrenia group ($271 496 vs $239 616, *P* = 0.0014). However, this difference was not statistically significant after adjustment (adjusted difference: $17 223, *P* = 0.075), suggesting that the cost difference was largely attributable to longer hospitalization and a higher comorbidity burden rather than schizophrenia per se.

### Post-operative complications

Post-operative infection rates were higher in the schizophrenia group in unadjusted analysis (2.9% vs 2.1%, *P* = 0.0080), but this difference did not persist after multivariable adjustment (adjusted OR 1.27, 95% CI 0.98–1.63, *P* = 0.0685). Rates of post-operative stroke (0.7% vs 0.7%, *P* = 0.9226), bleeding (0.2% vs 0.4%, *P* = 0.2957), and cardiovascular complications (0.2% vs 0.3%, *P* = 0.3837) did not differ significantly between groups in unadjusted or adjusted analyses. Thrombosis was lower in the schizophrenia group (0.0% vs 0.3%, *P* = 0.0167).

### Discharge disposition

Discharge patterns differed markedly between groups. Schizophrenia patients were significantly less likely to be discharged home without services (24.4% vs 37.3%, adjusted OR 0.38, *P* < 0.0001) or to home health care (34.4% vs 38.6%, adjusted OR 0.56, *P* < 0.001). Conversely, they were substantially more likely to require discharge to skilled nursing facilities or intermediate care facilities (37.6% vs 20.8%, adjusted OR 1.74, 95% CI 1.33–2.27, *P* < 0.0001). No patients with schizophrenia left against medical advice, compared to 0.1% of non-schizophrenia patients.

## Discussion

This large-scale, nationally representative analysis suggests that schizophrenia is associated with increased in-hospital mortality, nearly two additional hospital days, and a 74% increase in the likelihood of requiring post-acute skilled nursing care following CABG. These associations persist after comprehensive adjustment for demographic, clinical, and hospital characteristics. Our results underscore critical disparities in perioperative care for patients with serious mental illness and highlight opportunities for targeted, integrated interventions.

### Mortality risk in context

The increased adjusted mortality odds observed in our study are directionally consistent with the broader literature, though several prior reports document larger effect sizes and important contextual differences that merit discussion. Attar *et al*, in a nationwide Danish registry study specifically examining CABG outcomes in patients with schizophrenia, reported a 50% increase in 30-day perioperative mortality, a larger effect than our adjusted OR of 1.30[12]. In the acute coronary syndrome context, a 2022 systematic review and meta-analysis by Chan *et al* demonstrated that schizophrenia was associated with heightened post-acute coronary syndrome mortality irrespective of incident status, and both schizophrenia and bipolar disorder patients had significantly lower revascularization rates[17]. A national Scottish retrospective cohort study by Pearsall *et al* reported 30-day mortality odds ratios of 1.95 (95% CI 1.64–2.30) for schizophrenia following myocardial infarction, alongside substantially lower revascularization rates (HR 0.57, 95% CI 0.48–0.67)[18]. Taken together, these studies indicate that the mortality and care-access disparities we observe in the CABG setting reflect a pattern consistently documented across coronary interventions and geographic settings. The more modest magnitude of our mortality estimate may reflect survivorship selection, as patients with schizophrenia who successfully reach CABG in the US healthcare system may represent a medically stable subset^[^12,17,18^]^. Our study extends this literature by providing contemporary, US-specific data for CABG specifically, using a nationally representative sample with comprehensive covariate adjustment. The borderline nature of the mortality association, combined with the absence of significance in unadjusted analysis, indicates that the finding should be interpreted cautiously and viewed as hypothesis-generating rather than definitive.

Multiple mechanisms may contribute to excess mortality. Patients with schizophrenia face systemic barriers to optimal perioperative care, including delayed presentation, diagnostic overshadowing (attribution of cardiac symptoms to psychiatric illness), communication difficulties, and fragmented care coordination^[^19,20^]^. Antipsychotic medications may increase surgical risk through metabolic effects, QT prolongation, or interactions with anesthetic agents[21]. Cognitive impairment associated with schizophrenia may affect comprehension of post-operative instructions and medication adherence. Social isolation and limited support networks may compromise discharge planning and recovery[22].

Regarding post-operative complications, rates of infection, stroke, bleeding, and cardiovascular complications were similar between groups after adjustment. Thrombosis was lower in the schizophrenia group (0.0% vs 0.3%, *P* = 0.0167); however, this finding should be interpreted cautiously, given the very low absolute event rates in both groups and the absence of thrombotic events in the schizophrenia cohort, which likely drove the statistically significant *P*-value.

### Prolonged hospitalization and resource utilization

The 1.74-day increase in length of stay, while modest in absolute terms, represents a 17.6% relative increase and has substantial implications for healthcare costs, resource allocation, and patient outcomes. Prolonged hospitalization increases the risk of hospital-acquired infections, delirium, deconditioning, and thromboembolism[23]. For patients with schizophrenia specifically, prolonged hospitalization may disrupt psychiatric medication regimens, trigger symptom exacerbations, and complicate care coordination with outpatient providers.

Multiple factors likely contribute to extended hospitalization. Challenges in post-operative pain management, psychiatric symptom management, and behavioral issues may delay medical stabilization. Discharge planning complexity is amplified by the need to coordinate cardiac rehabilitation, psychiatric follow-up, and social services. Many patients with schizophrenia face housing instability or inadequate home support, necessitating alternative discharge arrangements[24]. The attenuation of the cost difference to non-significance after adjustment (adjusted difference: $17 223, *P* = 0.075) suggests that hospitalization duration and comorbidity burden, rather than schizophrenia per se, largely explain the higher unadjusted charges.

### Discharge disposition and post-acute care needs

Perhaps most striking is the 74% increased likelihood of requiring skilled nursing facility placement. Only 24.4% of schizophrenia patients achieved routine home discharge compared to 37.3% of non-schizophrenia patients. This disparity persists despite the schizophrenia cohort being 7 years younger on average, when younger age typically predicts better recovery and home discharge.

This finding reflects multiple interrelated factors: functional limitations, cognitive impairment, inadequate home support, housing instability, and psychiatric symptom severity. Skilled nursing facilities may be ill-equipped to manage complex psychiatric needs alongside cardiac recovery, potentially compromising both psychiatric and cardiac outcomes. Alternative models, including enhanced home health services with integrated psychiatric care, transitional care programs, and specialized cardiac rehabilitation with psychiatric consultation, merit investigation as potentially more effective and patient-centered approaches^[^25,26^]^.

### Cardiovascular risk factor profile

The cardiovascular risk profile in our schizophrenia cohort merits discussion. Higher rates of diabetes (58.0% vs 51.1%) and tobacco use (41.6% vs 18.4%) align with well-documented associations between schizophrenia and these modifiable risk factors^[^27,28^]^. Antipsychotic-induced metabolic syndrome contributes substantially to diabetes risk, while tobacco use remains highly prevalent in schizophrenia due to complex neurobiological, psychological, and social factors[29]. The paradoxically lower rates of hypertension, dyslipidemia, and atrial fibrillation may reflect diagnostic under-recognition, differential healthcare access, or competing mortality before CABG candidacy.

The younger mean age (59.6 vs 66.3 years) of schizophrenia patients undergoing CABG warrants consideration. This may reflect accelerated cardiovascular aging due to psychiatric illness and its treatments, or survivorship bias, whereby patients with schizophrenia who survive long enough to reach CABG candidacy represent a select, medically stable subset. Prior research documents premature cardiovascular disease onset in schizophrenia, with myocardial infarction occurring 10–15 years earlier than in the general population[30].

### Clinical and policy implications

Our findings suggest several actionable strategies to improve outcomes:

First, preoperative optimization should address modifiable psychiatric and medical risk factors. Psychiatric consultation before elective CABG can optimize medication regimens, assess cognitive function, identify psychosocial barriers, and establish post-operative psychiatric care plans. Smoking cessation and diabetes management warrant intensified focus, given their prevalence in this population.

Second, perioperative protocols should be adapted for serious mental illness. This includes psychiatry involvement in surgical care teams, proactive delirium prevention strategies, careful medication reconciliation with attention to drug–drug interactions, and enhanced nursing education on managing behavioral symptoms while respecting patient dignity.

Third, discharge planning must begin early and address psychiatric, medical, and social needs comprehensively. Social work involvement, coordination with community mental health providers, housing assessment, and caregiver engagement are critical. Transitional care models with psychiatric-medical integration show promise and warrant evaluation in this population[31].

Fourth, from a health systems perspective, these findings argue for integrated care models that address medical and psychiatric needs concurrently rather than sequentially. Medical-psychiatric units, consultation-liaison psychiatry services, and collaborative care programs may improve outcomes and reduce disparities[32].

### Strengths and limitations

This study’s strengths include a large sample size, national representation, robust statistical methods with comprehensive covariate adjustment, and the examination of multiple clinically relevant outcomes. The NIS provides generalizable insights into real-world care delivery and outcomes across diverse hospital settings.

Several limitations warrant consideration. As an administrative database, the NIS relies on ICD-10-CM coding and lacks clinical detail, including schizophrenia illness severity, antipsychotic medication type and duration, psychiatric treatment adherence, social support, and surgical complexity. Residual confounding is, therefore, possible. The NIS does not capture long-term outcomes beyond hospital discharge, and selection bias may exist if patients with schizophrenia who undergo CABG represent a medically stable subset. The 2017–2020 study period overlaps with the COVID-19 pandemic, which may have influenced care delivery. All findings should be interpreted within the context of these constraints.

## Conclusions

Schizophrenia was associated with increased odds of in-hospital mortality (adjusted OR 1.30, 95% CI 1.001–1.69, *P* = 0.049); this association was of marginal statistical significance and should be considered hypothesis-generating. Schizophrenia was also associated with nearly two additional hospital days and a 74% increased likelihood of skilled nursing facility discharge following CABG. These findings persist despite the schizophrenia cohort being approximately seven years younger and after comprehensive risk adjustment. Addressing these disparities requires integrated care models that address psychiatric and medical needs concurrently, optimized preoperative preparation, adapted perioperative protocols, and proactive multidisciplinary discharge planning. Future research should evaluate targeted interventions to ensure equitable access to high-quality cardiac surgical care for all patients, regardless of psychiatric comorbidity.

## Data Availability

All data obtained in the current study are available from the corresponding author upon reasonable request.

## References

[1] Laursen TM Nordentoft M Mortensen PB. Excess early mortality in schizophrenia. Annu Rev Clin Psychol 2014;10:425–48. 10.1146/annurev-clinpsy-032813-153657 24313570

[2] Hjorthøj C Stürup AE McGrath JJ. Years of potential life lost and life expectancy in schizophrenia: a systematic review and meta-analysis. Lancet Psychiatry 2017;4:295–301. 10.1016/S2215-0366(17)30078-0 28237639

[3] Walker ER McGee RE Druss BG. Mortality in mental disorders and global disease burden implications: a systematic review and meta-analysis. JAMA Psychiatry 2015;72:334–41. 10.1001/jamapsychiatry.2014.2502 PMC4461039 25671328

[4] Correll CU Solmi M Veronese N. Prevalence, incidence and mortality from cardiovascular disease in patients with pooled and specific severe mental illness: a large-scale meta-analysis of 3,211,768 patients and 113,383,368 controls. World Psychiatry 2017;16:163–80. 10.1002/wps.20420 PMC5428179 28498599

[5] Olfson M Gerhard T Huang C. Premature mortality among adults with schizophrenia in the United States. JAMA Psychiatry 2015;72:1172–81. 10.1001/jamapsychiatry.2015.1737 26509694

[6] Foley DL Morley KI. Systematic review of early cardiometabolic outcomes of the first treated episode of psychosis. Arch Gen Psychiatry 2011;68:609–16. 10.1001/archgenpsychiatry.2011.2 21300937

[7] De Hert M Detraux J van Winkel R. Metabolic and cardiovascular adverse effects associated with antipsychotic drugs. Nat Rev Endocrinol 2011;8:114–26. 10.1038/nrendo.2011.156 22009159

[8] Druss BG Bradford WD Rosenheck RA. Quality of medical care and excess mortality in older patients with mental disorders. Arch Gen Psychiatry 2001;58:565–72. 10.1001/archpsyc.58.6.565 11386985

[9] Druss BG Bradford DW Rosenheck RA. Mental disorders and use of cardiovascular procedures after myocardial infarction. JAMA 2000;283:506–11. 10.1001/jama.283.4.506 10659877

[10] Kisely S Smith M Lawrence D. Inequitable access for mentally ill patients to some medically necessary procedures. CMAJ 2007;176:779–84. 10.1503/cmaj.060482 PMC1808525 17353530

[11] Protty MB Lacey A Smith D. Increased morbidity, mortality and length of in-hospital stay for patients with acute coronary syndrome with pre-morbid psychiatric diagnoses. Int J Cardiol 2017;236:5–8. 10.1016/j.ijcard.2017.01.067 28111056

[12] Attar R Berg S Friberg Ö. The impact of schizophrenia on major perioperative outcomes after coronary artery bypass surgery: a nationwide population-based study. Eur Heart J 2017;3:198–202.

[13] Koomen EM Hutten BA Schut AFC. Psychiatric disorders and adverse outcomes in patients with cardiovascular disease. J Affect Disord 2021;280:388–96.

[14] Tully PJ Baker RA. Depression, anxiety, and cardiac morbidity outcomes after coronary artery bypass surgery: a contemporary and practical review. J Geriatr Cardiol 2012;9:197–208. 10.3724/SP.J.1263.2011.12221 PMC3418911 22916068

[15] Rymaszewska J Kiejna A Hadrys T. Depression and anxiety in coronary artery bypass grafting patients. Eur J Psychiatry 2003;18:155–60. 10.1016/s0924-9338(03)00052-x 12814847

[16] Agha RA Mathew G Rashid R. Transparency In The reporting of Artificial Intelligence the TITAN guideline. Prem J Sci 2025;10:100082.

[17] Chan JKN Chu RST Hung C. Mortality, revascularization, and cardioprotective pharmacotherapy after acute coronary syndrome in patients with severe mental illness: a systematic review and meta-analysis. Schizophr Bull 2022;48:981–98. 10.1093/schbul/sbac070 PMC9434477 35786737

[18] Pearsall R Smith DJ Pelosi A. Severe mental illness and mortality and coronary revascularisation following a myocardial infarction: a retrospective cohort study. BMC Med 2021;19:67. 10.1186/s12916-021-01937-2 PMC7983231 33745445

[19] Jones S Howard L Thornicroft G. ‘Diagnostic overshadowing’: worse physical health care for people with mental illness. Acta Psychiatr Scand 2008;118:169–71. 10.1111/j.1600-0447.2008.01211.x 18699951

[20] Mitchell AJ Malone D Doebbeling CC. Quality of medical care for people with and without comorbid mental illness and substance misuse: systematic review of comparative studies. Br J Psychiatry 2009;194:491–99. 10.1192/bjp.bp.107.045732 19478286

[21] Huyse FJ Touw DJ van Schijndel RS. Psychotropic drugs and the perioperative period: a proposal for a guideline in elective surgery. Psychosomatics 2006;47:8–22. 10.1176/appi.psy.47.1.8 16384803

[22] Cohen CI Meesters PD Zhao J. New perspectives on schizophrenia in later life: implications for treatment, policy, and research. Lancet Psychiatry 2015;2:340–50. 10.1016/S2215-0366(15)00003-6 26360087

[23] Engel HJ Tatebe S Alonzo PB. Physical therapist–established intensive care unit early mobilization program: quality improvement project for critical care at the University of California San Francisco Medical Center. Phys Ther 2013;93:1319–1327. 10.2522/ptj.20110420 23559525

[24] Folsom DP Hawthorne W Lindamer L. Prevalence and risk factors for homelessness and utilization of mental health services among 10,340 patients with serious mental illness in a large public mental health system. Amer J Psychiatry 2005;162:370–76. 10.1176/appi.ajp.162.2.370 15677603

[25] Naylor MD Brooten DA Campbell RL. Transitional care of older adults hospitalized with heart failure: a randomized, controlled trial. J Am Geriatr Soc 2004;52:675–84. 10.1111/j.1532-5415.2004.52202.x 15086645

[26] Druss BG von Esenwein SA Compton MT. Budget impact and sustainability of medical care management for persons with serious mental illnesses. Amer J Psychiatry 2011;168:1171–78. 10.1176/appi.ajp.2011.11010071 PMC3775649 21676993

[27] Holt RI Peveler RC Byrne CD. Schizophrenia, the metabolic syndrome and diabetes. Diabetic Med 2004;21:515–23. 10.1111/j.1464-5491.2004.01199.x 15154933

[28] de Leon J Diaz FJ. A meta-analysis of worldwide studies demonstrates an association between schizophrenia and tobacco smoking behaviors. Schizophr Res 2005;76:135–57. 10.1016/j.schres.2005.02.010 15949648

[29] Winterer G. Why do patients with schizophrenia smoke? Curr Opin Psychiatry 2010;23:112–19. 10.1097/YCO.0b013e3283366643 20051860

[30] Goff DC Sullivan LM McEvoy JP. A comparison of ten-year cardiac risk estimates in schizophrenia patients from the CATIE study and matched controls. Schizophr Res 2005;80:45–53. 10.1016/j.schres.2005.08.010 16198088

[31] Unützer J Katon W Callahan CM. Collaborative care management of late-life depression in the primary care setting: a randomized controlled trial. JAMA 2002;288:2836–45. 10.1001/jama.288.22.2836 12472325

[32] Kathol RG Butler M McAlpine DD. Barriers to physical health care for patients with mental disorders: a review and quantitative analysis. Psychosom Med 2010;72:724–31. 10.1097/PSY.0b013e3181e2c4a0 20498293

